# Neurofilament Light Chain (NF-L) Stimulates Lipid Peroxidation to Neuronal Membrane through Microglia-Derived Ferritin Heavy Chain (FTH) Secretion

**DOI:** 10.1155/2022/3938940

**Published:** 2022-03-24

**Authors:** Li Gong, Qiuyue Yu, Haichao Wang, Chenhaoyi Xu, Yunxiao Dou, Bingjie Mao, Yanxin Zhao

**Affiliations:** Department of Neurology, Shanghai Tenth People's Hospital, Tongji University, Shanghai, China

## Abstract

A part of the axonal cytoskeleton protein complex, neurofilament light chain (NF-L) has been suggested as a pathological hallmark in various neurological disorders, including hemorrhagic stroke, vascular dementia, and cerebral small vessel disease. Neuroaxonal debris are mainly engulfed and phagocytosed by microglia, while the effects of NF-L on microglia have not been elucidated. Ferritin heavy chain (FTH) not only reflects the age-related status of microglia but may also be secreted into the extracellular space. After treatment of microglia with varying concentrations of NF-L (0-3 *μ*g/ml), we found robust increases in the number of secretory FTH-containing exosomes in the medium. Induction of the FTH-containing exosomes secreted from microglia stimulates neuronal loss and membrane lipid peroxidation, as assessed by CKK8 and C11-Bodipy^581/591^, respectively. However, this oxidative stress damage was attenuated by blocking *Fth1* expression. Our results suggest that NF-L, as a biomarker of axonal injury itself, could participate in neuronal ferroptosis in a nonclassical manner by secreting FTH-containing exosomes from microglia into the extracellular matrix.

## 1. Introduction

Neurofilament light chain (NF-L) is a member of the family of filament proteins and forms the cytoskeleton of neurons. The protein correlates with various neuronal injuries due to multiple neurodegenerative disorders, as its levels are proportionally elevated with extended neuroaxonal damage [1–3]. Recently, many studies have indicated that NF-L in the cerebrospinal fluid (CSF) and bloodstream may serve as a reliable biomarker for the progression of age-related neurological disorders, including cerebral small vessel disease (CSVD) [4–6]. Classical biomarkers for neurodegenerative conditions such as the *β*-amyloid or phosphorylated tau proteins are involved in the pathogenesis of Alzheimer's disease (AD), including the formation of senile plaques and nerve fiber entanglement, respectively [7, 8]. However, at present, most studies only assess the association of NF-L with the progression or outcome of neurodegenerative disorders. Whether protein is involved in the pathogenesis of those diseases has not yet been elucidated.

Microglia are ubiquitously distributed cells in the central nervous system (CNS) and play a vital role in immunity and surveillance in the human brain [9]. Recent *in vivo/vitro* studies illustrated that neuroaxonal debris could be engulfed, phagocytosed, and degraded by HLA-DR-expressing microglia [10–12]. This clearance process could further lead to proliferation of microglia and an immunity disorder. In the CNS, the major iron stores are found within microglia and modulated by ferritin heavy chain (FTH), which also closely reflects the aging status of microglia [13]. For instance, clinical evidence indicates that CSF levels of FTH not only reflect iron status in the brain but also have clinical relevance in predicting outcomes in age-related neurodegeneration diseases [14, 15]. Moreover, rather than maintaining iron homeostasis within cells, ferritin can be released from microglia/macrophages through a nonclassical secretory pathway, supporting survival of other brain cell types or inducing cell death [16, 17]. Here, in culture, we examined the influence of NF-L on the secretory process of microglia-derived FTH, and the association of FTH in the extracellular space with lipid peroxidation of the neuronal membrane. Our results suggested that elevated NF-L levels could lead to increased amounts of FTH-containing exosomes in the extracellular matrix, consequently participating in neuronal ferroptosis.

## 2. Materials and Methods

### 2.1. Microglia and Neuronal Cultures

As previously described by our published study [9], BV-2 cells (a microglia cell line) were obtained and maintained. Primary cortical neurons were extracted from Sprague-Dawley (SD) fetal rats and seeded on poly-L-lysine-coated culture plates in Neurobasal Medium (Thermo Fisher Scientific) supplemented with 2% B27 (Gibco), 1% GlutaMAX (Gibco) in a 5% CO_2_ incubator at 37°C. Half of the culture medium was replaced every 2–3 days.

### 2.2. Neurofilament Light Chain (NF-L) Treatment

BV-2 microglia cells were treated with increasing concentrations of NF-L (Abcam), from 0 to 3 *μ*g/ml. After 48 h of treatment, the microglia supernatant was collected to extract FTH-containing exosomes, which were identified by transmission electron microscopy, CD81 staining, and ELISA. Finally, cortical neurons were cultured for 24 h with variable concentrations of FTH-containing exosomes extracted from microglia. Similarly, the microglia were divided into two groups, based on the presence or absence of *Fth1* mRNA. Then, after 48 h of treatment of microglia with 0 and 3 *μ*g/ml NF-L, FTH-containing exosomes from microglia medium were mixed with cortical neurons for 24 h.

### 2.3. Enzyme-Linked Immunosorbent Assay (ELISA)

Microglia were seeded on 96-well plates. Following treatment of microglia with different concentrations of NF-L for 48 h, the supernatant was collected, and FTH levels were determined using an Fth1 ELISA Kit (Abcam).

### 2.4. Quantitative Real-Time PCR

As our work previously described [9], total RNA extraction and cDNA synthesis were performed using TRIzol reagent (Invitrogen) and reverse transcription kit (Takara) following the manufacturers' instructions. The specific primers used for PCR are as follows: actin F-5′-GGCTGTATTCCCCTCCATCG-3′, R-5′-CCAGTTGGTAACAATGCCATGT-3′ and Fth1 F-5′-CAAGTGCGCCAGAACTACCA-3′, R-5′-GCCACATCATCTCGGTCAAAA-3′. Quantitative real-time PCR was performed using SYBR FAST qPCR Master Mix (KAPA) with appropriate TaqMan primers and the ABI PRISM 7900HT Sequence Detection System. The detection was performed in triplicate.

### 2.5. Cell Viability Evaluation

Cell viability was determined by cell counting kit-8 (CCK8) according to the manufacturer's instructions. Inoculate cell suspensions (100 *μ*l/well) were seeded in a 96-well plate. Plates were preincubated in a humidified incubator (37°C, 5% CO_2_). Different concentrations of substances to be tested were added to the plates, and the plates were placed in the incubator for 48 h. CCK8 solution was added to each well for 2 h. Then, the absorbance was measured at 450 nm using a BIO-TEK Elx-800 microplate reader.

### 2.6. Exosome Isolation and Identification

BV-2 microglia cells were treated with increasing concentrations of NF-L (0 to 3 *μ*g/ml) for 48 h. Culture supernatants were centrifuged at 4°C at 2000 × g for 20 min and 10,000 × g for 30 min prior to ultracentrifugation at 100,000 × g for 70 min at 4°C. Pellets were resuspended in cold PBS and centrifuged again at 100,000 × g for 60 min. The morphology of exosomes was detected by transmission electron microscopy (normal size: 30-150 nm), and the exosomal marker transmembrane protein (CD81) was quantified by western blotting.

### 2.7. Transmission Electron Microscopy (TEM)

For exosome TEM observation, exosomes extracted from culture supernatant of BV-2 microglia were fixed with 2% uranyl-acetate solution for 1 minute on electron-microscope grids. After blotting excess fluid and drying the grid for 10 minutes, exosomes were imaged with a TEM at 80 kV.

### 2.8. siRNA Transfection

Cells were transfected with nontargeting siRNA (negative control) or special siRNA targeting rat Fth1 (5′-CCGAGAAACTGATGAAGCT-3′) designed by GenePharma using Lipofectamine™ 3000 reagent following the manufacturer's instructions. Media were refreshed 6 h later, and cells were harvested 48 h later.

### 2.9. Neuronal Lipid Peroxide Assessment

To visualize the neuronal lipid oxidation, cells were seeded in 8-well plates and incubated for 30 min at 37°C with C11-BODIPY^581/591^ (2 *μ*M) in growth media in dark. Images were taken with a TCSNT confocal laser scanning system (Leica). The green and red fluorescence of C11-BODIPY^581/591^ was acquired simultaneously using double wavelength excitation (488 and 568 nm) and detection (emission bandpass filters 530/30 and 590/30).

### 2.10. Immunofluorescence Microscopy

After treatment with variable concentrations of NF-L (0 to 3 *μ*g/ml), BV-2 cells were fixed in 4% paraformaldehyde and permeabilized in 0.2% Triton. Slides were blocked in 5% BSA and incubated in primary antibodies against Fth1 (ABclonal) and LC3B (CST), both diluted 1 : 200 with 5% BSA, overnight at 4°C. The next day, cells were washed in PBS and incubated in secondary antibody Alexa Fluor® 488 AffiniPure Donkey Anti-Rabbit IgG (Jackson ImmunoResearch) and Cy™3 AffiniPure Donkey Anti-Mouse IgG (Jackson ImmunoResearch) for 1 h at room temperature. Finally, slides were imaged on a confocal laser scanning microscope (Leica).

### 2.11. Statistical Analysis

Data are presented as mean ± standard deviation for continuous variables. Student's *t*-test was used to compare the normally distributed quantitative variables. Statistical analyses were completed using GraphPad software Inc. Prism Version 8, US. *p* < 0.05 was considered statistically significant.

## 3. Results

### 3.1. Association of NFL Treatment with Secretion of Ferritin Heavy Chain (FTH) from Microglia

**Figure 1:**
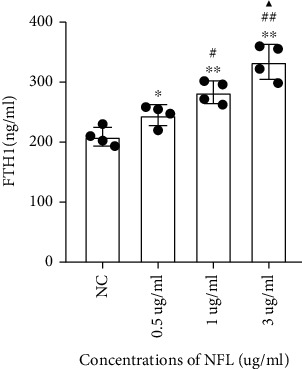
Effects of NF-L treatments on the changes in FTH-containing exosome from microglia. Microglia was treated with an increasing dose of NF-L for 48 h after which the levels of FTH-containing exosome were analyzed by ELISA. A significant increase in the level of FTH-containing exosome (*p* < 0.01) is observed above 0.5 *μ*g/ml NF-L compared with control. Values represent mean ± SEM (*n* = 4). ^∗^*p* < 0.05 and ^∗∗^*p* < 0.01 as compared with control; ^#^*p* < 0.05 and ^##^*p* < 0.01 as compared with the 0.5 *μ*g/ml NF-L treatment group; ^▲^*p* < 0.05 as compared with the 1 *μ*g/ml NF-L treatment group.

To ascertain whether NF-L treatment resulted in FTH secretion, we first extracted and evaluated the levels of FTH-containing exosomes in media following NF-L treatment of microglia. Morphological identification was performed by transmission electron microscopy, and CD81 was verified by western blotting. Considering that NF-L is present at a concentration of approximately 3 *μ*g/ml in the brain, the NF-L concentration was maintained at or below this level. Microglia cell viability after treatment with 0.5, 1, and 3 *μ*g/ml of NF-L was reduced significantly in comparison to the control, as assessed by CCK8 assay (Sup. 1). However, significantly increased levels of FTH-containing exosomes (*p* < 0.01) were found when treated with 0.5, 1, and 3 *μ*g/ml of NF-L, as assessed by ELISA (Figure 1). Due to secretory autophagy, the other pathway mediating ferritin secretion, we further evaluated whether the increase in NF-L was related to FTH-containing autophagosomes within the microglia. Microglia treated with increasing NF-L concentrations for 48 hours showed elevated intensities of FTH-containing autophagosomes, as measured by immunofluorescence (Sup. 2).

### 3.2. FTH-Containing Exosome Results in Neuronal Loss and Membrane Oxidation

**Figure 2:**
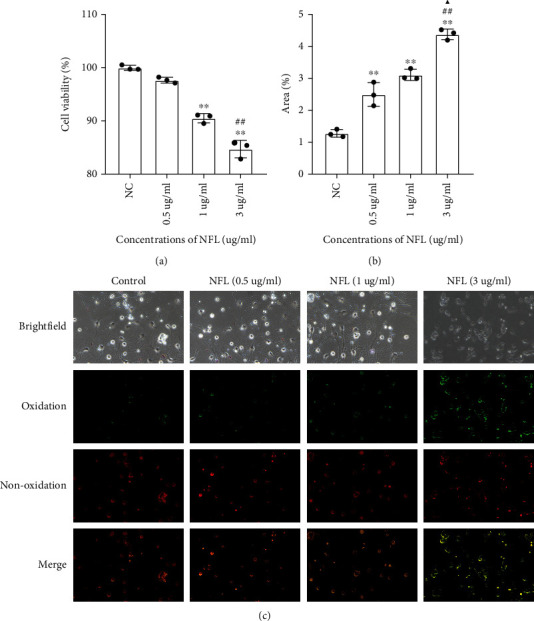
Effects of FTH-containing exosome on neuronal viability and lipid peroxidation. Cortical neuron was treated with 24 h FTH-containing exosome, after which the cell viability of cortical neurons decreased as measured by CCK8, and the lipid peroxidation levels of neuronal membrane increased as evaluated by C11-Bodipy^581/591^. Relative quantification of cell viability (a) and area percentage of C11-Bodipy^581/591^ (b), respectively; the representative images of C11-Bodipy^581/591^ (c). Values represent mean ± SEM (*n* = 3). ^∗∗^*p* < 0.01 as compared with control; ^##^*p* < 0.01 as compared with the 0.5 *μ*g/ml NF-L treatment group; ^▲^*p* < 0.05 as compared with the 1 *μ*g/ml NF-L treatment group.

To investigate the effects of FTH-containing exosomes on neuronal activity and lipid peroxidation, exosomes were extracted from microglia cell supernatant after treatment with increasing doses of NF-L. Then, cortical neurons were treated for 24 h with FTH-containing exosomes, which decreased the activity of cortical neurons, as measured by CCK8 (Figure 2(a)), and increased the lipid peroxidation levels of the neuronal membrane, as evaluated by C11-Bodipy^581/591^ (Figures 2(b) and 2(c)).

### 3.3. Knockdown of FTH1 mRNA in Microglia Reduces Formation of FTH-Containing Exosome and Rescues Oxidative Injury of Neuronal Membrane

**Figure 3:**
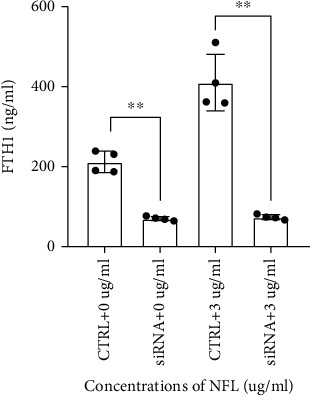
NF-L treatments of microglia induce changes in the levels of FTH-containing exosome in the presence and absence of *FTH1* mediated by siRNA. After 0 *μ*g/ml and 3 *μ*g/ml NFL interventions of microglia for 48 h, changes in extracellular FTH-containing exosome were determined by ELISA and CD81. The panels represent relative quantification of secretory FTH. Values represent mean ± SEM (*n* = 4), ^∗∗^*p* < 0.01.

**Figure 4:**
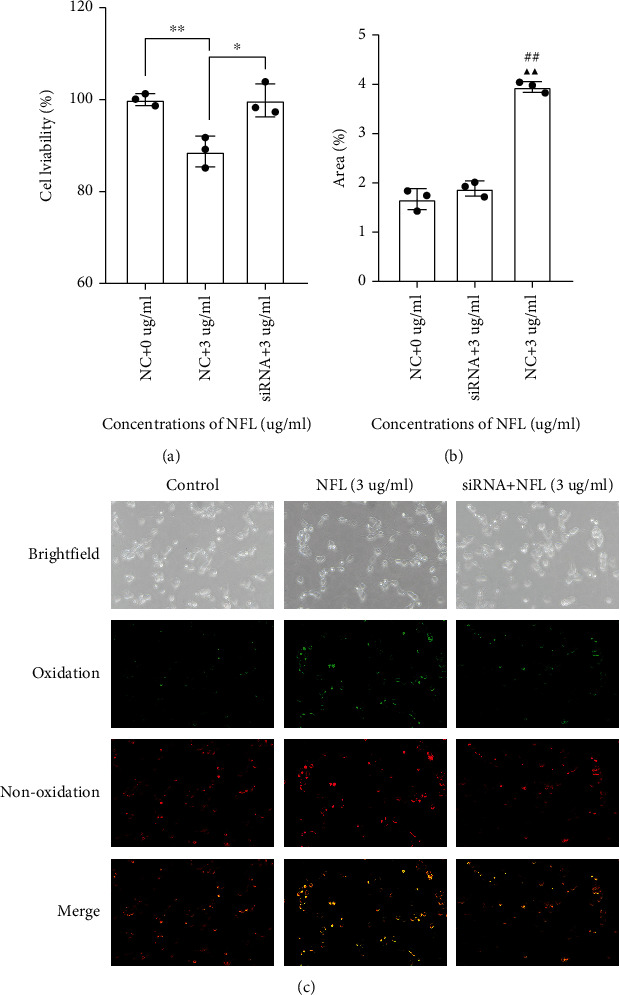
Influence of FTH-containing exosome on neuronal viability and lipid peroxidation in the presence and absence of *FTH1* mediated by siRNA. FTH-containing exosomes were extracted from culture supernatant of BV-2 microglia with 3 *μ*g/ml NFL intervention for 48 h. Then, cortical neuron was treated with the secretory FTH for 24 h in the presence and absence of *FTH1* mediated by siRNA. Relative quantification of cell viability (a) and area percentage of C11-Bodipy^581/591^ (b), respectively; the representative images of C11-Bodipy^581/591^ (c). Values represent mean ± SEM (*n* = 3). ^∗^*p* < 0.05 and ^∗∗^*p* < 0.01; ^##^*p* < 0.01 as compared with the 0 *μ*g/ml NF-L treatment group; ^▲▲^*p* < 0.01 as compared with the 3 *μ*g/ml NF-L treatment group in the absence of *FTH1*.

To explore the mechanism by which NF-L affected lipid peroxidation levels of neurons through FTH-containing exosomes from microglia, microglia cells were divided into two groups according to whether *Fth1* expression was inhibited or not. The knockdown efficiency of siRNA was verified by qPCR, and *Fth1* mRNA in microglia was reduced by more than 80% after knockdown (Sup. 3). Results indicated a significant decrease in FTH levels in exosomes from microglia in the group treated with *Fth1* siRNA as compared to the group treated with nontargeting siRNA (Figure 3). Furthermore, the FTH-containing exosomes from microglia in both groups interfered with cortical neurons. Results showed that cell viability of cortical neurons in the *Fth1* knockdown group was significantly higher than that of the group expressing *Fth1* (Figure 4(a)), while the lipid peroxidation level of neurons was significantly lower than that of the *Fth1-*expressing group (Figures 4(b) and 4(c)).

## 4. Discussion

NF-L has been recognized widely as a biomarker of multiple neurological disorders, such as hemorrhagic stroke and cerebral small vessel disease. The protein not only reflects the severity of neuronal injury but also predicts disease transition from early to late stage [18, 19]. For example, in the animal model of multiple sclerosis, NF-L was surrounded and phagocytized by microglia and associated with glia activation [10, 11]. However, the influence of NF-L on microglia, in turn, has not been elucidated. In the present study, our results first suggested that increased NF-L levels promote ferritin secretion from microglia by the exosome-mediated pathway. Then, the secreted ferritin in the supernatant leads to oxidative damage of the neuronal membrane. Finally, we knocked down *Fth1* with siRNA to investigate the potential mechanism by which NF-L affected the secretion of ferritin exosomes from microglia.

Cellular iron trafficking and detoxification are mainly dependent on the functions of ferritin, a 450 kDa protein complex consisting of 24 subunits of light and heavy chains [20]. Serving as a ferroxidase, producing ferric iron within the core of the ferritin protein, ferritin heavy chain (FTH) has long been considered an intracellular iron storage protein [21]. However, recent evidence revealed two potential mechanisms underlying the secretion of ferritin including exosome-mediated and autophagy-mediated pathways [22, 23]. Exosomes are a group of small secretory vesicles, composed of membrane-encased structures 40–100 nm in size. Recent evidence suggested that ferritin containing the majority of iron stored usually existed within the peripheral cells, such as macrophages, while the secretion of iron-load ferritin might be modulated through the exosome mechanism [23]. However, the secretion of ferritin is mainly concentrated in peripheral cells, such as macrophages. Further evidence showed that oligodendrocytes could release FTH through an unconventional secretion route involving exosomes. Here, we revealed that microglia could secrete FTH through the exosome-mediated pathway, suggesting that the secretion of ferritin is concentrated not only in peripheral cells but also in microglia cells. Therefore, our finding supports and extends those previous results.

In addition to the exosome mechanism, recent studies revealed that autophagy might also contribute to ferritin transport [21]. Indeed, we identified FTH was highly expressed in autophagosomes marked with LC3B beyond the extracellular exosome, indicating the ability of autophagosomes to release FTH. However, whether ferritin can be secreted from the cell by secretory autophagy or can be damaged by autophagy lysosomes may be primarily based on differences in the SNARE system [24, 25]. Thus, further studies are needed to address this pathway in microglia.

Iron accumulation has been associated with a majority of neurodegenerative diseases such as Alzheimer's disease (AD); it is important to investigate whether ferritin exosomes participate in iron-mediated neuronal death pathways, such as ferroptosis, characterized by membrane lipid peroxidation. Although ferritin is primarily considered an intracellular iron storage protein, it has been found in the CSF and peripheral blood of patients with AD, and its levels in CSF predict AD outcomes [14, 26, 27]. The role of microglia-secreted ferritin has not yet been elucidated, but the release and transfer of ferritin from macrophages to oligodendrocyte precursor cells (NG2 cells) have been reported, supporting the possibility of intercellular ferritin transfer within the central nervous system [16]. In line with this concept, we now provide evidence that NF-L could be a part of the ferroptosis source through secreting FTH-containing exosomes from microglia. Several factors might be related to this finding. First, changes in the iron storage function of ferritin, FTH/FTL ratio, have been demonstrated in neurodegeneration diseases [28]. When FTH increases, it can result in instability of ferritin to store iron and the presence of more highly oxidative Fe^2+^ [29]. Second, another study revealed that carcinoma cells could have unique drug tolerance due to ferroptosis resistance, driven by an exosome-ferritin pathway to transport iron out of the cell [30]. Therefore, these findings might explain why the viability of microglia did not differ with elevated levels of NF-L treatment. It was assumed that microglia could protect against NF-L treatment by producing more ferritin-binding intracellular free iron and transferring more ferritins to the extracellular space in the form of ferritin exosomes. However, the self-protection behavior of microglia might lead to the extracellular iron dyshomeostasis and subsequent neuronal ferroptosis.

In contrast to these findings, a recent study found that oligodendrocytes secrete FTH1 through extracellular vesicles to protect against iron-mediated ferroptotic axonal damage and form a part of the neuronal antioxidative stress system [31]. Ferritin in the CNS has additional functions compared to the periphery, where ferritin mainly serves as a storage protein. Under trophic conditions, microglia are myelination-supporting cells that secrete FTH as a source of iron for oligodendrocytes, maintaining their survival [17, 32, 33]. However, iron-enriched medium from microglial culture also causes increased oligodendrocyte cytotoxicity [17], suggesting that the influence of microglia on the survival or death of other CNS cells may partially depend on their iron status. In summary, under physiological conditions, microglia secrete ferritin as a trophic factor to adjacent cells, which may, in turn, exert adverse effects on the surrounding environment, including possibly causing pathology.

In conclusion, the present study provides novel insights into the possibility that NF-L plays an important role in the secretory process of microglia-derived ferritin, including the shedding of FTH, an oxidative factor which could damage the neuronal membrane. Although neuroaxonal debris must be taken up for clearance, microglia might play an adverse role through augmenting oxidative damage to the neuronal membrane. This relationship between NF-L and microglia could underlie the concurrence of ferritin and NF-L as putative biomarkers of neurodegenerative diseases. The relevance of our results on secretory microglia-derived FTH to the neuronal ferroptosis pathway *in vitro* requires further *in vivo* studies. Although the medium containing increased levels of FTH-containing exosomes leads to severe neuronal damage that is rescued by neuronal *FTH1* knockdown, microglia-derived exosomes might contain other cytotoxic factors such as *β*-amyloid. Therefore, further studies are required to establish whether this FTH-secreting pathway is shared and how FTH is exported, including in exosomes or by a secretory autophagy pathway, as well as where and how FTH induces lipid peroxidation in neuronal membranes.

## Data Availability

The data used to support the findings of this study are available from the corresponding author.

## Supplementary Materials

Supplementary 1 Sup 1: effects of NFL treatments on the microglia cell viability assessed by CCK8 assay.

Supplementary 2 Sup 2: effects of NFL treatments on the changes in FTH-containing autophagosome marked with LC3B.

Supplementary 3 Sup 3: the knockdown efficiency of siRNA for FTH1 mRNA in microglia.
