# Supplementary figures and images for: Neurofilament Light Chain (NF-L) Stimulates Lipid Peroxidation to Neuronal Membrane through Microglia-Derived Ferritin Heavy Chain (FTH) Secretion
Source: Oxid Med Cell Longev. 2022 Mar 24;2022:3938940. doi: 10.1155/2022/3938940 (PMC8972155; doi:10.1155/2022/3938940)

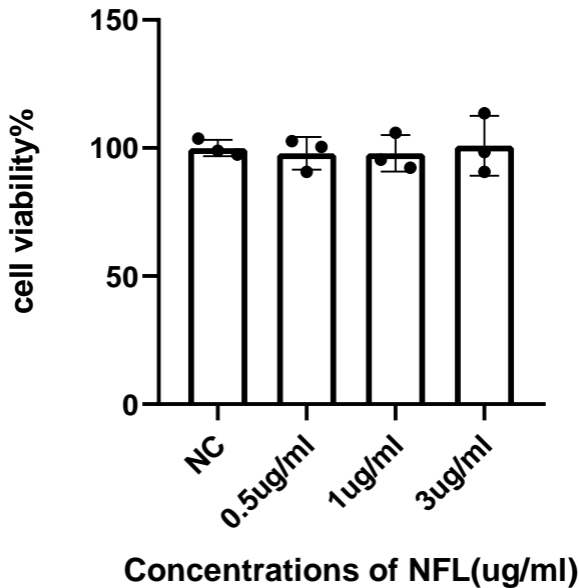

Supplement: Supplementary 1 — Sup 1: effects of NFL treatments on the microglia cell viability assessed by CCK8 assay. [file 3938940.f1.pdf]

Control

NFL(0.5ug/ml)

NFL(1ug/ml)

NFL(3ug/ml)

Fth1

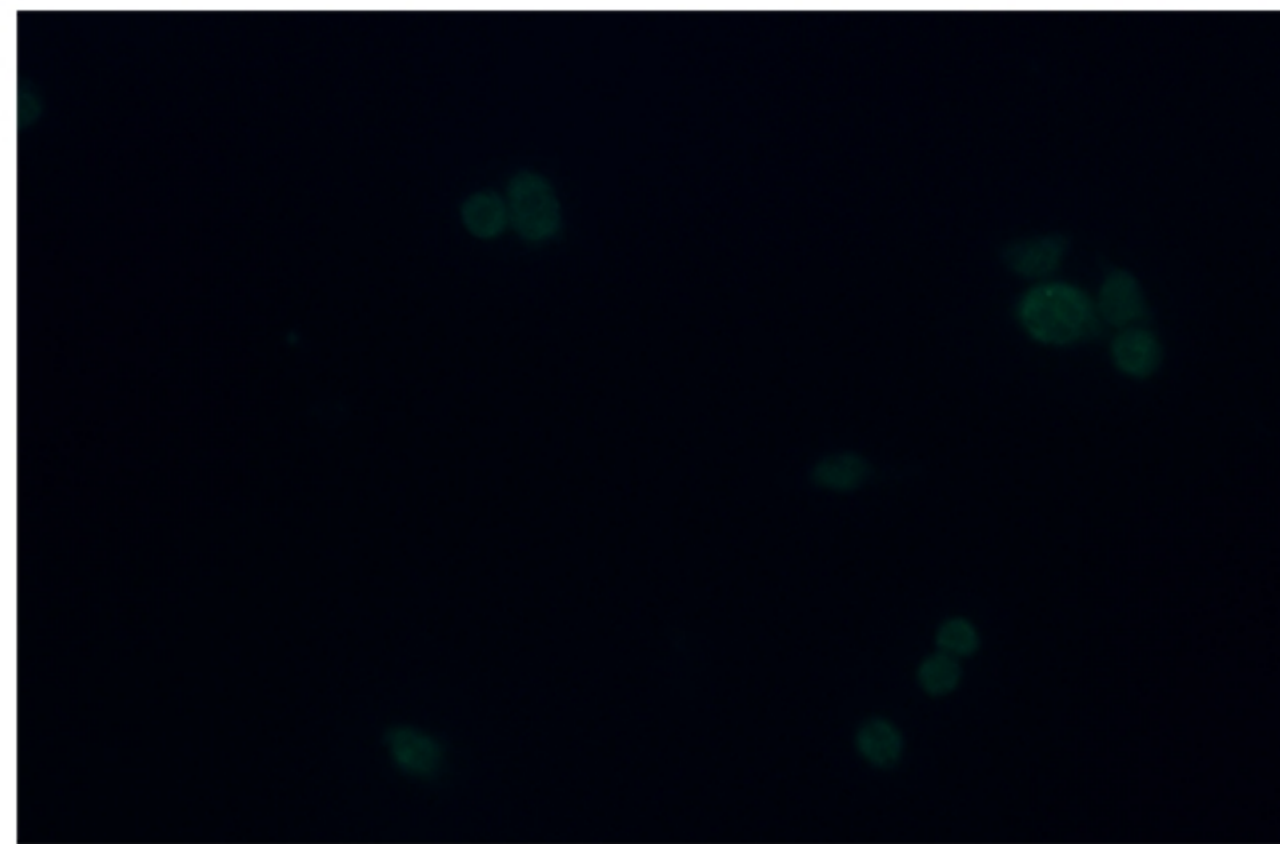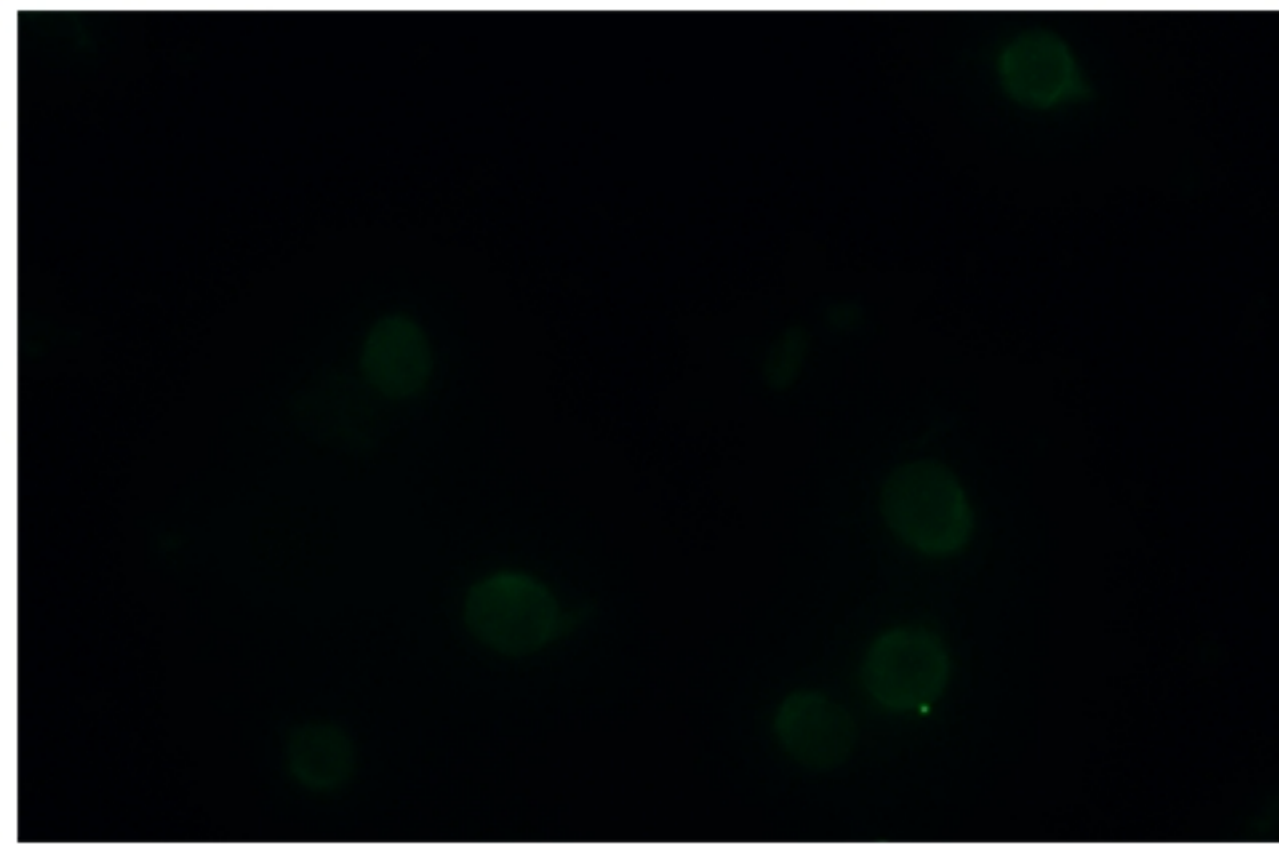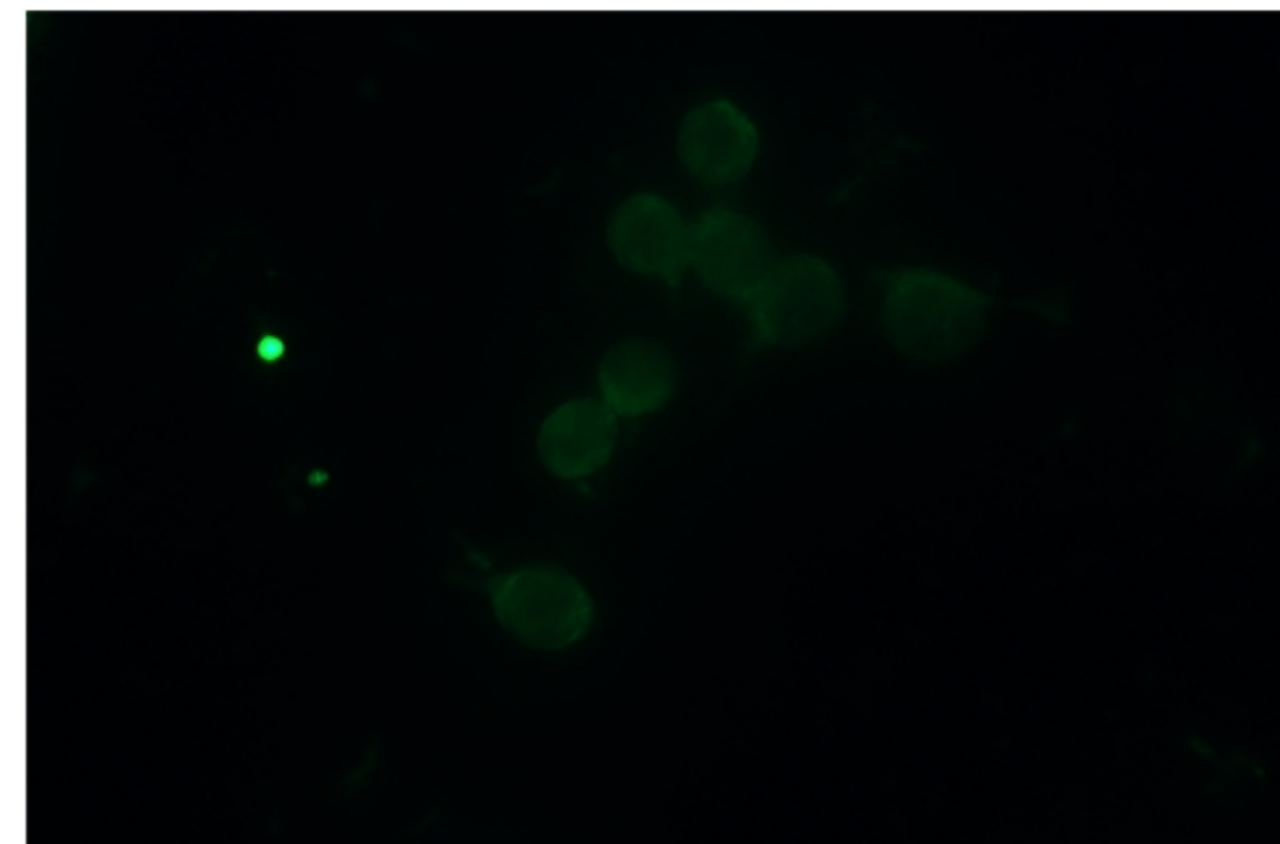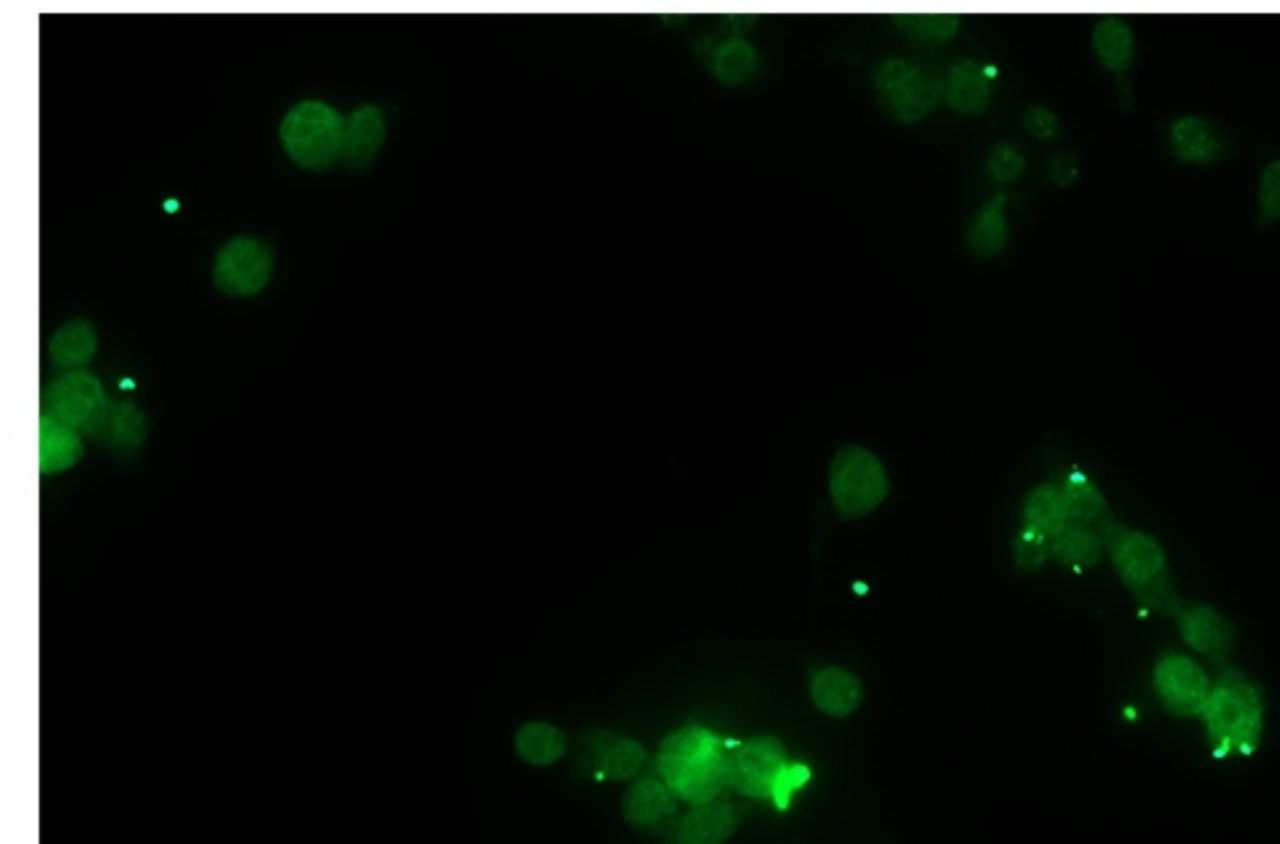

LC3B

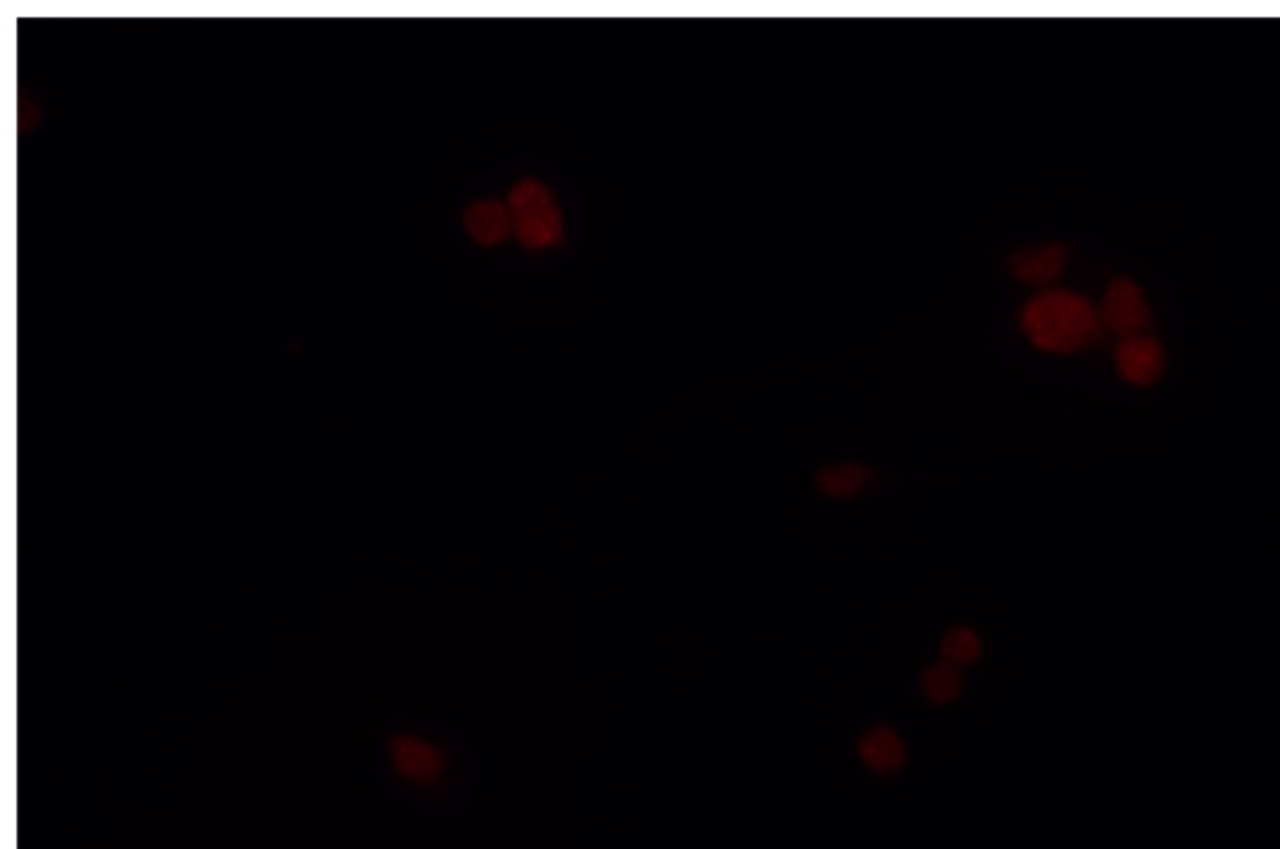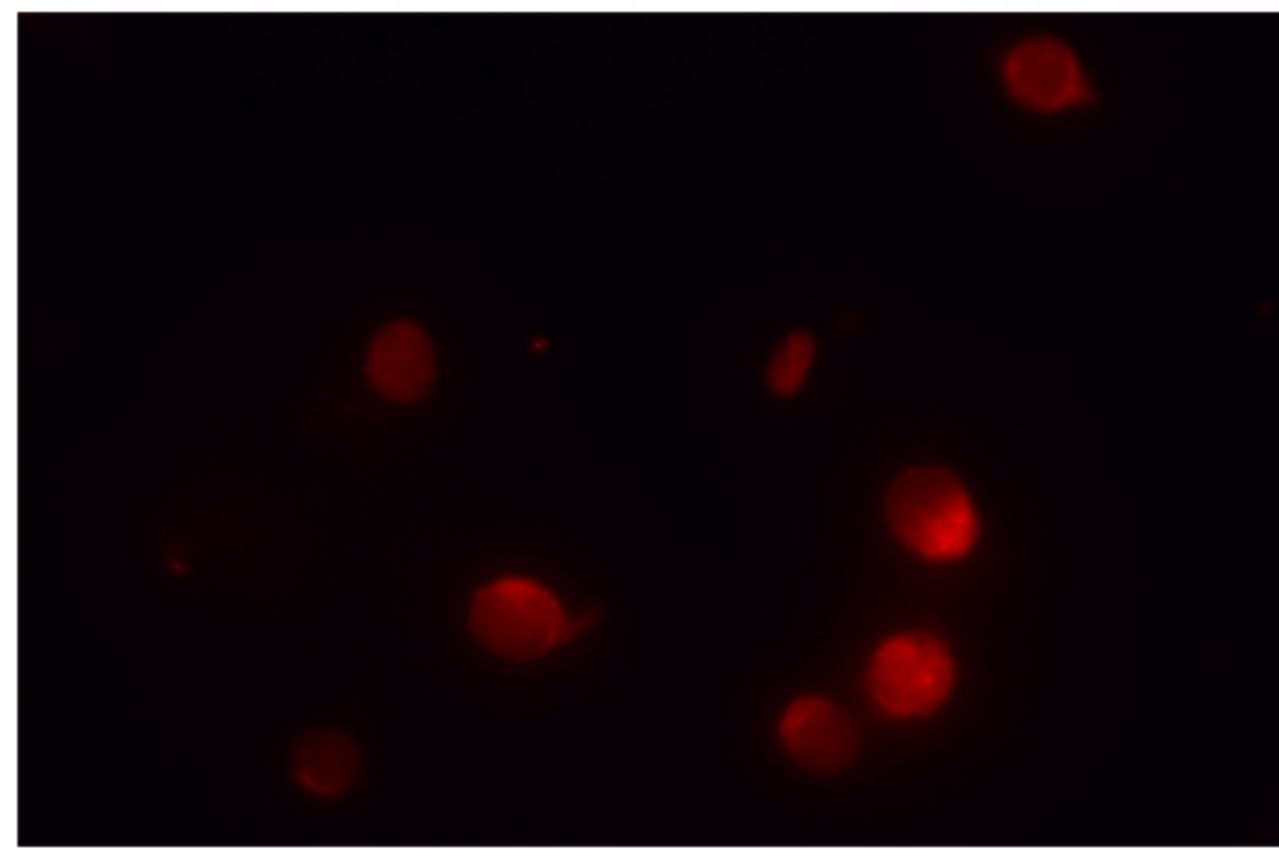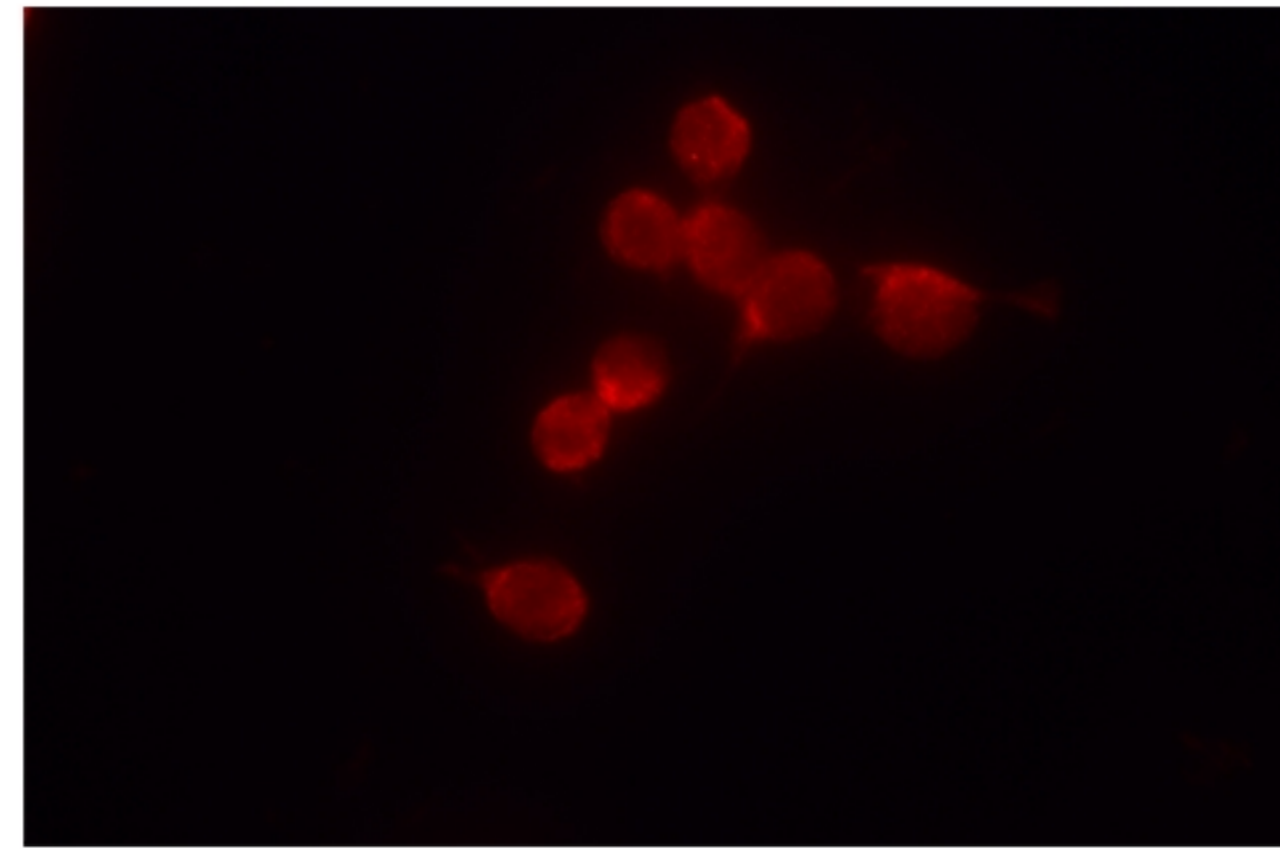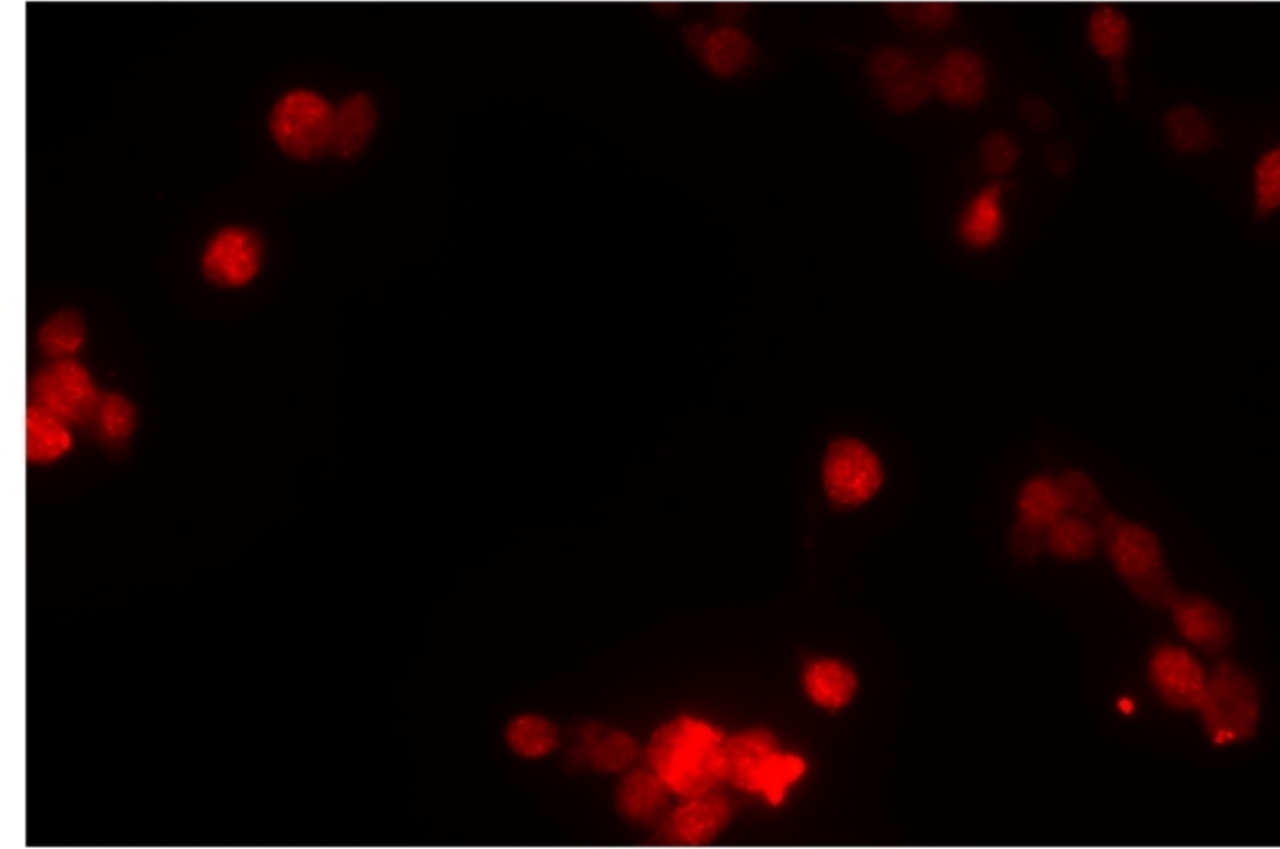

Supplement: Supplementary 2 — Sup 2: effects of NFL treatments on the changes in FTH-containing autophagosome marked with LC3B. [file 3938940.f2.pdf]

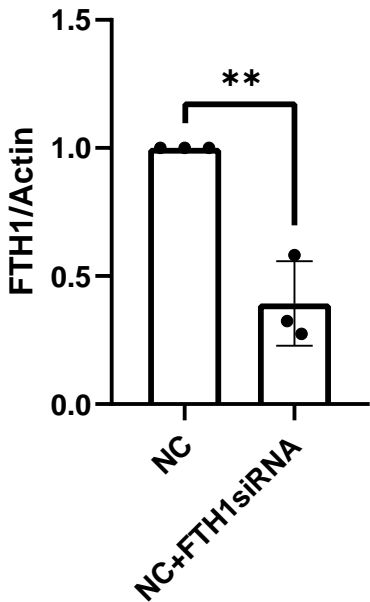

Supplement: Supplementary 3 — Sup 3: the knockdown efficiency of siRNA for FTH1 mRNA in microglia. [file 3938940.f3.pdf]
